# Proceedings of the 13th international transplantation symposia: mTOR-inhibition: what have we learned and how so we best apply the learning

**DOI:** 10.1186/s13737-015-0027-7

**Published:** 2015-12-22

**Authors:** Alan G. Jardine

**Affiliations:** University of Glasgow, Glasgow, G12 8QQ UK

In *Transplantation Research* we have recently published a series of articles on the role of mammalian Target of Rapamycin (mTOR) inhibitors [1–3]. This is a new departure for the Journal, on which we hope to build in the future, of publishing online conference supplements. The articles are based on the most recent of a long-running annual conference, supported initially by Wyeth and subsequently by Pfizer, following the introduction of sirolimus (Rapamune). For me, this meeting gave delegates a unique experience, and I doubt if any participant failed to learn something to change their clinical practice. Using a combination of didactic lectures, workshops and interactive sessions for around 300 clinicians and 20–30 faculty members, the design of the meeting promoted dialogue and exchange of information. Perhaps the key aspect of the meeting was to mix faculty, including some of the leading figures in transplantation, with transplant clinicians from a range of backgrounds in an informal atmosphere. Initially, the faculty members were those involved in the development of sirolimus (SRL) and early clinical trials, but later involved those with interests in infection, cardiovascular disease and cancer where there were specific issues or opportunities relating to the use of mTOR-inhibitors; and subsequently, to the developing role of mTOR on cellular function and cancer, intravascular stents, and other areas. The focus of the meeting also evolved with time: initially devoted to SRL, in later meetings this agent became the fulcrum for a meeting that covered other emerging immunosuppressants – including mTOR-inhibitors, such as everolimus, novel agents such as belatacept, and much broader aspects of transplant biology and management.

The long story of the discovery of SRL in the Easter Islands (hence the name Rapamune) to its introduction into clinical practice, is a fascinating tale [4, 5] – which has been reported previously – and which owes its development to Suren Sehgal, whose perseverance at Wyeth kept the development going; to clinician-scientists like Randy Morris who used it in an experimental setting, to the clinicians who led the early human trials. Immediately following its introduction, the aspiration was that SRL would offer an alternative to calcineurin inhibition (CNI), and avoid the adverse consequences of CNI on renal function. However, with time, it became apparent that – for the majority of patients – SRL did not offer comparable immunosuppression to CNI and, that the side effect profile was complex and sometimes difficult to manage. Around this time the Symphony trial [6] established the efficacy of a relatively simple regimen using tacrolimus (Tac), mycophenolate mofetil (MMF), and corticosteroids (CS), which deterred clinicians – particularly those outwith academic centres – from using novel agents. Subsequently, the place of SRL has become more focussed: to use in patients where CNI use has compromised graft function, in those with malignancy or at high risk of malignancy, and in the management of patients with – or at high risk – of viral infections such as CMV or BK nephropathy. Currently, while SRL is used in < 10 % of renal transplant recipients RTR), for those who tolerate the agent well it offers advantages with regard to long-term graft function, improved blood pressure control with reduced antihypertensive medication, and reduced risk of viral infections and malignancy [1–3].

In this edition, Ed Giessler, a leading basic scientist in experimental and human transplantation, reviews the role of mTOR-inhibitors in cancer [1, 7]. Successful transplantation is associated with an increase in the risk of malignancy, most marked for skin tumours and particularly squamous cell carcinoma (SCC) and the aggressive, cutaneous Merkel cell cancer – as a consequence of immunosuppression. Registry data show that half of all transplant recipients will develop some form of cancer during their lifetime [1, 7]. It is difficult to have imagined, even with hindsight, that mTORi would emerge as effective treatments for cancer, given the negative impact of previous agents on malignancy. However, various mTORi – including derivatives of SRL, such as temsirolimus – are now licenced for the treatment of a variety of tumours including renal cancers and angiomyolipomas (AML) associated with Tuberous Sclerosis Complex (TSC). The first inkling of their efficacy came in the demonstration that switching to SRL could regress Kaposi’s sarcoma (1,7), later studies – highlighted in this article have shown benefits of SRL in preventing the recurrence of common skin cancers in RTR. In this, and other reviews, the key role of mTOR in cell proliferation has been explained in more detail [1, 7].

In the second article, Helio Tedesco-Silva and colleagues review 15 years of experience of SRL in Sau Paulo, Brazil; one of – if not the – largest kidney transplant centre in the world. The size of their transplant population allowed this centre, in collaboration with others, to lead a number of early phase trials, and to study the possible benefits of *de novo* low-dose CNI-SRL combinations and to use the combination of SRL and Tac (when the latter agent emerged as the CNI of choice, 6), and to study SRL in combination with induction therapy using IL-2 receptor blockade. The results, in studies involving many hundreds of patients, show that it is possible to achieve similar acute rejection rates, and comparable graft function with SRL-CNI vs standard therapy but that even in the most experienced centres, withdrawal due to adverse side effects is higher with SRL, as is the incidence of clinically significant proteinuria. However, in this centre –as observed elsewhere – the incidence of CMV infection was halved in patients receiving SRL.

This centre was also involved in trials, following on from the experience with *de novo* use above, to investigate the use of a pre-determined switch from CNI to SRL. Over the years, there have been a series of studies examining the potential benefits of switching at time points from a few days to several months after transplantation [2, 3]. The findings of Tedesco-Silva *et al*., [2] mirror those of other studies. Patients with minimal proteinuria and good graft function, who tolerate SRL, did very well with better graft function than those who continued on conventional CNI based therapy. However, switching was associated with a higher incidence of acute rejection (albeit with limited long term effects), proteinuria and withdrawal due to intolerance.

These observations are consistent with more general experience that SRL-based immunosuppression is associated with very good graft function, but that its use is difficult in clinical practice. “Therapeutic inertia” on the part of both clinicians and patients has limited the use of SRL for this reason, unless there is a driving clinical indication – most commonly intolerance of CNI, or the risk or presence of malignancy, or viral infections (CMV and BK virus; 3).

In the third article, Fritz Diekmann and colleagues [3] from Barcelona, present their extensive clinical experience with SRL. Covering similar ground to the review above [2], and providing an accessible table of the key studies, they highlight the difficulties in switch protocols – from CNI to mTOR-inhibitors – and the difficulties with “reactive” switches in clinical practice, particularly when patients have significant proteinuria. In spite of this, they see a place for SRL based therapy in patients with CNI cosmetic side effects and neurotoxicity, patients with or at risk of BK and CMV infection and cancers, particularly those with a viral aetiology. Perhaps the most important contribution of this group has been to inform and help patients, and clinicians, to manage side effects including mouth ulcers and skin rashes, to allow patients to continue with SRL in the longer term and the importance of targeting lower trough levels.

There are few new agents for transplant immunosuppression, either in development or that have come into clinical practice since the introduction of SRL. The difficulty in developing new agents for a relatively small therapeutic indication and market, and the limitations of the conventional composite trial endpoint – acute rejection, graft loss, death and loss to follow-up – have deterred pharmaceutical companies from investment in this area. The recent history of clinical trials has been early discontinuation of clinical development programmes (e.g. FTY-720 and AEB-071), and only belatacept has been licenced for clinical use, with limited impact. Although transplant outcomes have improved dramatically in the last 40 years, the agents we use have toxicity that would be unacceptable in other clinical areas, and most agents require close monitoring of drug levels. The SRL “story” outlined in the articles in this supplement illustrates how the indications for SRL use have evolved with time as we have understood the mechanisms of action, and moved to lower dose and target levels – a “story” that is not dissimilar to the other agents in clinical use. For SRL, it now appears that *de novo* use is impractical in most centres, and that its place is to minimise or replace CNI in patients with CNI related toxicity; and those with or at risk of viral infections or malignancy. These indications limit use to 5–10 % of the total transplant population; a subgroup for whom it is the best agent, with a key role. However, there are large regional variations. SRL may be favoured in regions where CMV seropositivity is high and the cost of universal prophylaxis prohibitive; and in regions where the rate of cancer is high, such as the countries in South East Asia with high levels of genitourinary malignancy. The development of SRL has taught us many things, not least the unexpected role of mTOR in cancer, and with the “retrospectroscope” one wonders if SRL had been developed first would we be asking ourselves whether to use CNI that are, perhaps, easier to use but are associated with an unacceptable risk of post-transplant malignancy?
